# Training Working Memory to Reduce Rumination

**DOI:** 10.1371/journal.pone.0090632

**Published:** 2014-03-03

**Authors:** Thomas Onraedt, Ernst H. W. Koster

**Affiliations:** Department of Experimental-Clinical and Health Psychology, Ghent University, Ghent, Belgium; University of Groningen, Netherlands

## Abstract

Cognitive symptoms of depression, such as rumination, have shown to be associated with deficits in working memory functioning. More precisely, the capacity to expel irrelevant negative information from working memory seems to be affected. Even though these associations have repeatedly been demonstrated, the nature and causal direction of this association is still unclear. Therefore, within an experimental design, we tried to manipulate working memory functioning of participants with heightened rumination scores in two similar experiments (n = 72 and n = 45) using a six day working memory training compared to active and passive control groups. Subsequently the effects on the processing of non-emotional and emotional information in working memory were monitored. In both experiments, performance during the training task significantly increased, but this performance gain did not transfer to the outcome working memory tasks or rumination and depression measures. Possible explanations for the failure to find transfer effects are discussed.

## Introduction

Depression is generally considered an invalidating mental disorder that is associated with major suffering for affected individuals and their direct social network [1]. Moreover, at the societal level depression poses a major challenge as the burden of this disorder is large in terms of sick leave [2], co-morbidity with health problems [3] and treatment costs [4]. Despite decades of research on psychological as well as pharmacological interventions, major challenges remain. These challenges include improving the response rates and ensuring stable levels of remission [5], [6]. Increasingly, translational research, based on specific knowledge of causal risk factors of depression research, is trying to develop new methods to specifically target the etiological and maintaining mechanisms of depression. In the present work we explore the utility of a new training methodology to improve cognitive impairments in the context of cognitive risk factors of depression.

### Cognitive impairments and depressive symptoms

Cognitive impairments are considered important features of depression. According to the diagnostic and statistical manual of mental disorders [1], these impairments include memory, concentration and attention problems. In recent years, it has been strongly advocated that some of the cognitive impairments observed in depression are more than simple correlates of negative mood but provide a major contribution to impaired emotion regulation, rumination, and persistent negative affect [7], [8]. This notion is based on extensive empirical research that shows that different aspects of cognitive control, such as inhibition, attentional control, and working memory capacity are impaired in individuals with clinical depression [9], [10].

In line with the idea that cognitive control is a causal risk factor for depression it has been found that such impairments can also be observed in samples that are not currently depressed but that have a high risk for depression. For instance Joormann et al. [11] demonstrated that, following a negative mood induction, never-disordered daughters of depressed mothers showed biased selective attention to negative emotional stimuli compared to a control group. Other studies used samples that have high levels of rumination, a key cognitive risk factor for depression. Rumination is defined as repetitive thoughts focused on the causes, symptoms and implications of one's negative mood state [12]. The most used self-report instrument to assess ruminative thinking is the Ruminative Response Scale (RRS) [13], measuring to what extent individuals generally respond in a ruminative way when feeling down, sad or depressed. Empirical studies have extensively demonstrated that rumination is associated with various aspects of depression risk [14]. Other studies found that individuals with high rumination scores are characterized by impaired inhibition and switching in working memory when negative information was in focal attention [15], [16]. Interestingly, impaired ability to switch attention away from negative information in working memory is prospectively associated with elevated rumination upon encountering stressful events [17]. Moreover, a prospective study in remitted depressed individuals showed that cognitive impairments in emotion processing predicted rumination and new depressive symptoms one year later [18].

It has been argued that many of the cognitive impairments described earlier can be mapped onto the functioning of working memory. Working memory can be defined as a limited-capacity system for the temporary storage of information and a mechanism of central or executive attention that regulates the content of the working memory [19]. It is thought that many of the impairments observed at the level of attention and memory can be explained by difficulties in expelling irrelevant or no longer relevant negative information from working memory [16], [20]. However, in the context of depression and rumination, many studies have also observed overall reduced working memory capacity [21], [22]. The latter finding is in line with the observation that depression is characterized by reduced levels of frontal activity [23]. Moreover, rumination has also been linked to neural difficulties switching from resting state to task-engagement [24].

### Causality

Even though the link between cognitive impairments and depressive symptoms has been demonstrated in several correlational and prospective studies, no clear inferences about the nature of this association can be made. It is possible that depression and rumination deplete working memory resources [25]. Alternatively, working memory impairments such as difficulties removing negative information out of working memory, may cause a prolonged and almost chronic exposure to these stimuli. Such difficulties to interrupt the vicious cycle of negative thoughts can be considered a key cognitive process underlying rumination [26]. Finally, in correlational and prospective designs it is also possible that a third factor influences both working memory and rumination or depressive symptoms. To investigate whether working memory processes have a causal influence on depressive symptoms, an experimental design is required. Using the cognitive bias modification methodology [27] to examine the functional role of cognitive processes, the expected causal factor, being working memory functioning, has to be manipulated to subsequently monitor the effects on rumination and subclinical depressive symptoms.

Currently, there is an extensive debate about the efficacy of working memory training and the transferability of training effects [28]–[31]. A major challenge of working memory training procedures is to obtain transfer of training to new tasks and contexts. In recent years, several studies have shown promising results using a working memory training paradigm. For instance Jaeggi et al. [32] used the dual n-back task to train working memory and found, next to improvements on the training task, considerable gains in fluid intelligence scores compared to a control group (see also [33]). However, these results have been challenged based on inappropriate designs (absence of an active control condition) and inappropriate transfer tasks that do not tap aspects of working memory [30].

Although the efficacy of working memory training in improving working memory performance in healthy individuals is still under debate, working memory training may have interesting effects in the context of psychopathology or traits that are characterized by reduced working memory performance [7], [34]–[36]. Owens et al. [37] were able to improve working memory capacity and filtering efficiency in a non-clinically depressed sample, using the dual n-back training. Moreover, these changes were associated with EEG measures before and after training. The results of these studies suggest that working memory training, and the dual n-back task in particular, might be a valid tool to manipulate working memory within an experimental design. Such training has interesting potential. If working memory training proves to cause sustainable beneficial effects, it could complement existing treatments or (relapse) prevention programs. The main advantages of this kind of computerized training are that an online version is easily accessible for a large target group, relative low costs and the possibility for the health care professional to monitor the day to day progress.

### Current study

The aim of the two recent experiments was to examine the effect of a well-documented non-emotional working memory training on the processing of non-emotional and emotional information in working memory and measures of rumination and eventually depression in a population with elevated rumination. Since rumination is characterized by impaired performance on working memory tasks [21] we sought to investigate whether working memory training could change working memory functioning in relation to emotional and non-emotional information and whether such training could subsequently reduce the tendency to ruminate. This lower tendency to ruminate could eventually lead to a decrease in levels of (subclinical) depressive symptoms.

In two experiments, participants were randomly allocated to a six-day dual n-back training, an active control condition or (only in experiment 1) a no training control group. In the first experiment this training phase was preceded and followed by the Running Span Task [38], a classic working memory task, the Internal Shift Task [39] that measures the shifting ability in working memory, using emotional stimuli, the Beck Depression Inventory-II (BDI-II) [40] and the Ruminative Responses Scale (RRS) [13]. In the second experiment, to examine transfer effects of training, the Internal Shift Task was replaced by the Operation Span Task, another classic working memory task, and the emotional 2 back task, that measures the influence of negative information on the updating capacity of working memory. In both experiments the BDI-II and RRS were administered a third time, two weeks after training as follow-up.

These designs allowed to investigate the effect of the experimental manipulation on changes in working memory performance, rumination scores, and depressive symptoms after one week of training and at follow up. Summarizing the previous research three main hypotheses were generated for this study.

First of all, in line with previous training studies [32], [37], we expect that in the dual n-back training group, general working memory performance, as measured by the classic working memory tasks will improve, relative to the active control or no training group. Second, we expect that improved working memory performance will ameliorate the processing of emotional information [15], [16] and therefore hypothesize that the interference of emotional stimuli will decrease in the dual n-back group compared to the control groups. Finally we expect that improved processing of emotional information will lead to a more adaptive coping with daily stressors and therefore hypothesize that rumination and potentially depression scores will decrease in the dual n-back group relative to the control groups.

## Experiment 1: Methods

### Design overview

**Figure 1:**
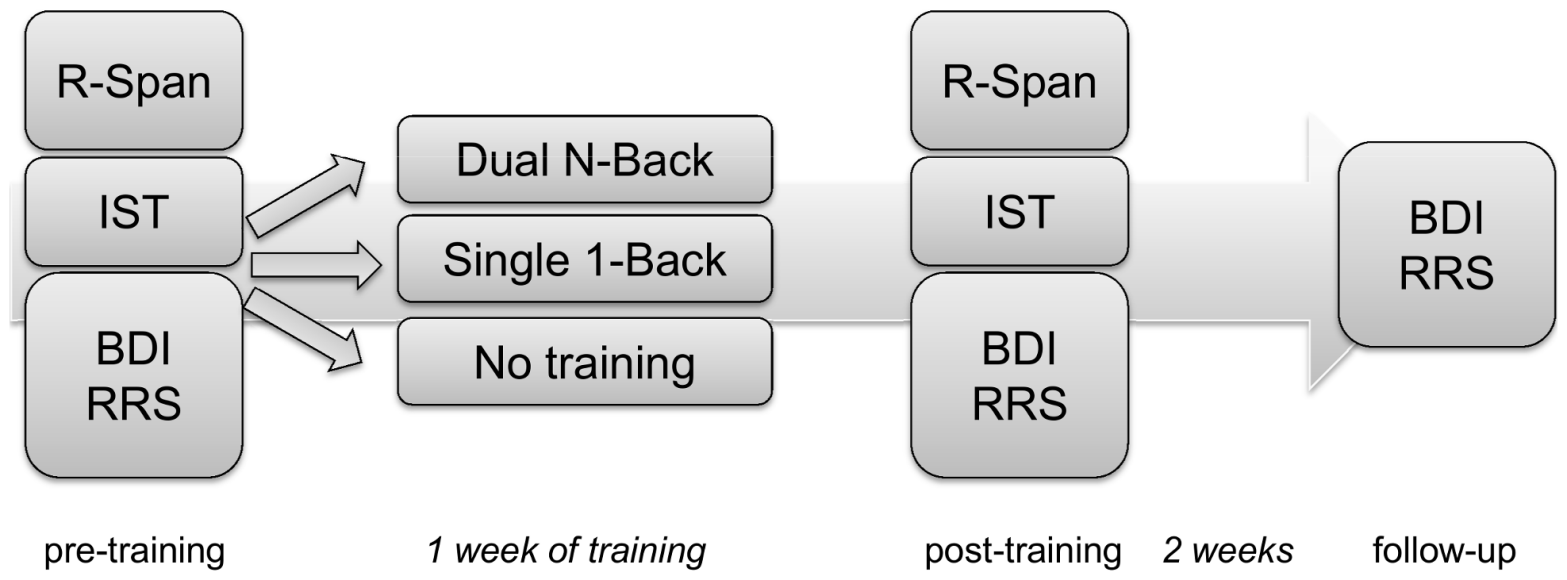
Design experiment 1.

An overview of the experiment design is presented in figure 1. At pre-training two working memory tasks, the Running Span Task and the Internal Shift Task were administered, followed by questionnaires. Subsequently, participants were randomly assigned to one of three homework training conditions. One week later the same computer tasks and questionnaires were administered at post-training. Another two weeks later, questionnaires were administered a third time during a follow-up.

### Participants

In this experiment, 79 undergraduates of Ghent University participated in return for a financial compensation (€ 25). The experiment was advertised through an online experiment managing system. Using a screening questionnaire, only participants with a RRS total score of 46 or above were invited for the experiment to ensure only individuals with a relative high tendency to ruminate could participate. Three participants were excluded because they did not show up for the posttest. Another four participants were excluded because they did not perform sufficient training sessions between both test moments. This resulted in a final sample of 72 participants. Participants' characteristics are presented in table 1.

**Table 1:** Group characteristics.

	Dual n-back	Single 1-back	No training
*N*	21	25	26
Age (*M* (*SD*))	20.19 (2.06)	20.92 (3.24)	19.85 (2.03)
Gender (M/F)	2/19	7/18	0/26

### Materials

#### Running memory Span Task (R-Span Task)

The R-Span Task was programmed by Nitz [41] in line with Broadway and Engle [38] and ran using the Inquisit software package [42] on a Pentium 4 computer with a 60 HZ 17 inch CRT monitor. In the R-Span Task, participants were instructed to report the last *n* letters, in the correct order, from a series of sequentially presented letters (a combination of F, H, J, K, L, N, P, Q, R, S, T or Y). The length of this series was variable, so participants could not predict when the series ended. The number of target letters varied from three to eight. Before each series participants were informed about the number of letters that had to be reported. Each letter was presented sequentially for 300 ms (with an interstimulus interval of 200 ms) in the center of a grey screen in a black 18 pt font. In total, 36 series of letters were presented, more precisely one block of six series for every target length (3, 4, 5, 6, 7 or 8). The order of these blocks was randomized. Within each block, the target letters were three times preceded by zero distractors, and one time by one, two and three letters, in a random order. After each series, participants could respond by clicking the recalled letters in the correct order on a 3×4 grid containing all possible letters. One point was assigned for every target letter that was correctly recalled and in the correct serial position.

#### Internal shift task (IST)

The IST [15], [39] was programmed using the E-prime software package [43], and ran on the same computer as the R-Span Task. In the IST, series of faces taken and adjusted from the Karolinska Directed Emotional Faces database [44] were sequentially presented at the center of a computer screen. The number of faces in each series varied randomly from 10 to 14. In the *emotional condition*, participants were instructed to keep a silent mental count of the number of neutral and angry faces. Therefore they had to keep two counters in mind that had to be updated every time a face was presented. Participants were instructed to press the space bar as soon as possible, every time they had updated their counters. Their reaction time was the main variable of interest. Two hundred ms after the key press the next face appeared. At the end of each series of faces, participants had to report how many neutral and angry faces they had seen. In the *non-emotional condition*, the same stimuli were used but participants had to keep a mental count of the number of male and female faces. The order in which both conditions had to be completed was counterbalanced across subjects. Based on the order of the faces, trials could be defined as shift or no-shift trials. A trial was considered a shift trial if the counter that had to be updated was different from the previous trial (e.g. angry-neutral or male-female). In a no-shift trial the counter that needed to be updated was the same as in the previous trial (e.g. neutral-neutral or male-male). By subtracting the mean reaction time of the shift trials from the mean reaction time of the no-shift trials, shift costs could be calculated separately for the emotional and non-emotional condition. In each condition, 12 series of faces were presented, preceded by three practice series.

#### Dual n-back training

For the homework training, participants installed the Brainworkshop software package [45] on their personal computer. Using this program, they could perform the dual n-back task [32]. In the dual n-back task, both visual and auditory information was presented sequentially at a rate of 3000 ms per trial (500 ms stimuli presentation, 2500 ms interstimuli-interval). In a 3×3 grid, a blue square appeared randomly in one of eight possible locations. Simultaneously, participants heard a spoken letter (C, H, K, L, Q, R, S or T). Every day the dual n-back task started at level *n* = 2. This means participants had to compare the current square position and letter with the square position and letter presented two steps before. Participants had to respond by pressing “Q” for matching positions and “L” for matching sounds. If both position and sound matched, both “Q” and “L” had to be pressed. If there was no match, no response had to be made. Each block included *n* + 20 combinations of a square and a letter, and comprised four visual matches, four auditory matches and two simultaneous (both visual and auditory) matches. If response accuracy within the block was 90% or greater, the dual n-back level was increased (e.g. if *n* = 3, current position and sound had to be compared with position and sound 3 steps before). If response accuracy was between 90 and 75%, dual n-back level was maintained, and if response accuracy was 75% or below, the dual n-back level was decreased. Participants in the dual n-back training group had to complete 20 blocks of 20 + *n* trials each day, during six days. This amount of training was shown to be sufficient to train working memory in subclinically depressed individuals [37].

#### Position and sound 1-back

Participants in the control training condition performed a single 1-back task, also using the Brainworkshop software package [45]. In contrast to the dual n-back task, they only had to monitor one stream of stimuli, more specifically the position of the square in the position 1-back condition or the spoken letter in the sound 1-back condition. Irrespective of performance, the *n* level was fixed at *n* = 1, so participants were instructed to compare the current stimulus with the previous one by pressing “Q” or “L” for a position or sound match respectively. Parallel to the dual n-back training condition, participants had to complete 20 blocks of 20 + 1 trials each day, during six days. The procedure of both control tasks was very similar to the dual n-back tasks, but in contrast to the dual n-back task, they only placed a minimal load on working memory capacities.

#### Questionnaires

In order to measure the presence and severity of depressive symptoms, the Dutch translation of the Beck Depression Inventory-II [40], [46] was used. The BDI-II is a 21 item (scored from 0 to 3) self-report measure and has been found to be a valid measure to establish depressive symptoms in both clinical and non-clinical samples [40], [47].

The Dutch translation of the Ruminative Response Scale [13], [48] was used to measure rumination. The Dutch version of the RRS is a 22 item self-report questionnaire that measures responses to a depressed mood that are repetitively focused on the self, symptoms or consequences of that depressed mood. Participants have to indicate how often they engage in these responses on a four-point Likert scale ranging from 1 (almost never) to 4 (almost always). Although a total score can be calculated, rumination as measured by the RRS cannot be seen as a unitary construct. An exploratory factor analysis of Treynor et al. [49] identified two distinct factors differentially related to depressive symptoms. The first factor, reflective pondering, consists of five items that assess the rather adaptive cognitive problem solving focused at improving one's mood and depressive symptoms. The second factor, ruminative brooding consists of five items that assess the maladaptive passive focus on the possible causes of their depressed mood. The RRS proved to be a reliable and valid measure of rumination [49].

#### Procedure

Participants were given limited instructions about the purpose of the study. They were told their participation consisted out of a six day homework computer task, preceded and followed by two computer tasks in the lab and some questionnaires. All participants provided their written informed consent to participate in this study. At pretest, the R-Span Task and IST were administered after completing the informed consent form. The BDI-II and RRS had to be completed the same day. Participants were randomly allocated to the dual n-back training condition, single 1-back control condition or no training condition. Within the single 1-back condition, participants were randomly allocated to either the position or the sound 1-back control training. Afterwards, the participants allocated to the position and sound 1-back condition were combined to one single 1-back control condition. After exactly one week of daily training, both computer tasks and questionnaires were administered at posttest. Finally, two weeks after posttest, the questionnaires were completed as follow-up. At the end of this follow-up, participants were debriefed about the purposes of this experiment. This experiment was approved by the ethics committee of the Faculty of Psychology and Educational Sciences, Ghent University.

#### Statistical analyses

Associations between computer task scores and questionnaires were inspected using Pearson correlations. To test for baseline differences between groups, a set of one way anovas was executed with group as independent variable. The effect of training was tested using a mixed anova design for each outcome variable, with time as within subject factor and group as between subject factor.

## Experiment 1: Results

### Baseline correlations

Correlations between the baseline computer tasks and questionnaires are presented in table 2. Running span score was negatively correlated with emotional shift cost, but not with non-emotional shift cost. Emotional and non-emotional shift cost were highly correlated. Running span score was negatively correlated with BDI-II score, but not with rumination scores, and only non-emotional shift cost was significantly correlated with ruminative brooding score. BDI-II score was significantly correlated with the RRS total and brooding score, but not with the reflection score. Finally, RRS total score was correlated with both RRS factors, but the brooding and reflection factor were not correlated.

**Table 2:** Pearson correlation between baseline variables.

	(1)	(2)	(3)	(4)	(5)	(6)	(7)
(1) R-Span Task score	-	−.297*	−.082	−.267*	−.046	−.024	−.109
(2) Emo. shift cost		-	.690^**^	.063	.085	.114	.146
(3) Non-emo. shift cost			-	.198	.100	.251*	.031
(4) BDI-II total				-	.295*	.235*	.055
(5) RRS total					-	.700^**^	.530^**^
(6) RRS brooding						-	.100
(7) RRS reflection							-

^*^ *p*<.05. ^**^ *p*<.01.

### Baseline group differences

One way anovas indicated that mean age, R-Span Task, IST, BDI-II and RRS scores were not significantly different between the dual n-back, single 1-back control, and no training control group (all *F*s<2.4) at pretest. There were however significant differences in gender ratio between conditions, χ^2^(2, *N* = 72) = 9.38, *p* = .009, see table 1.

### Dual n-back training performance

**Figure 2:**
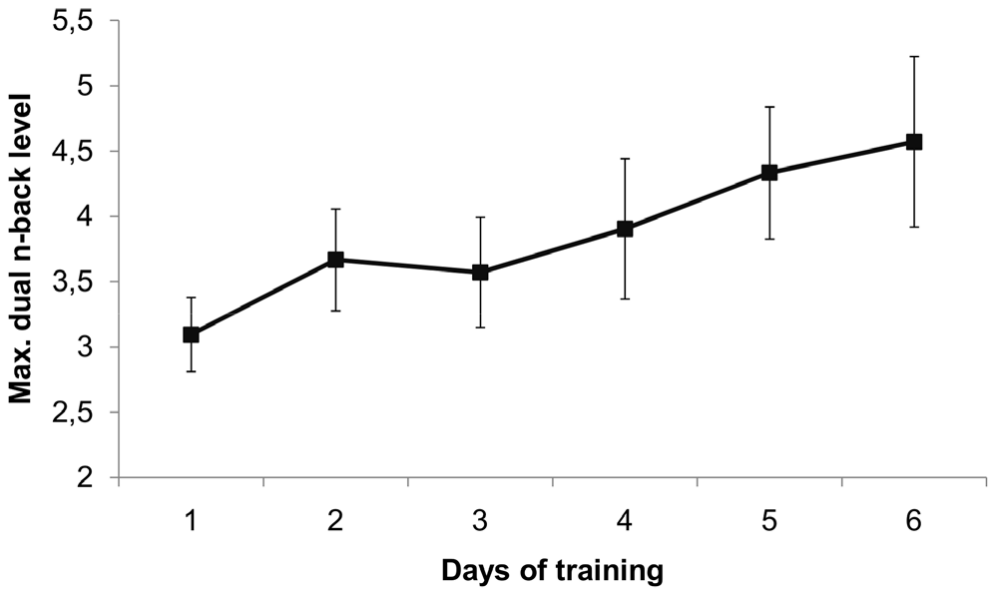
Dual n-back progress with 95% confidence intervals for experiment 1.

During the six day training, the performance of the adaptive dual n-back training group improved significantly. The average daily maximum *n*-level increased gradually from 3.10 (*SD* = 0.63) at day one to 4.50 (*SD* = 1.43) at day six, *t*(19) = 4.92, *p*<.001, Cohen's *d* = 1.10 (see figure 2). The increase in maximum n-level was not significantly correlated with the difference scores (post- minus pre-training score) for the R-Span, IST and RRS, all *p*'s >.29. There was however a marginally significant correlation between dual n-back improvement and BDI-II difference score, *r* = −.375, *p* = .093, indicating larger training gains are marginally associated with a smaller increase, or larger decrease in BDI-II score.

### Analyses of transfer effects

All task and questionnaires score are presented in table 3. Sound 1-back and position 1-back participants were collapsed after data exploration indicated no differences for all analyses.

**Table 3:** Computer task and questionnaire scores for T1, T2 and T3.

	Pre-training (T1)			Post-training (T2)			Follow-up (T3)		
	Dual n-back	Single 1-back	No training	Dual n-back	Single 1-back	No training	Dual n-back	Single 1-back	No training
R-span	20.62 (6.91)	21.36 (5.72)	19.35 (4.12)	22.95 (5.30)	22.72 (6.18)	20.08 (4.54)	-	-	-
IST							-	-	-
Emo. shift cost (ms)	403 (243)	386 (215)	498 (199)	382 (273)	376 (229)	431 (190)	-	-	-
Non-emo. shift cost (ms)	409 (238)	373 (192)	458 (196)	325 (195)	332 (203)	405 (216)	-	-	-
BDI-II	9.14 (8.74)	10.48 (8.99)	9.58 (7.36)	8.90 (8.41)	9.12 (7.65)	8.81 (7.04)	10.00 (10.44)	8.84 (7.74)	10.38 (7.76)
RRS total	49.33 (8.99)	47.12 (11.16)	47.88 (9.84)	48.43 (10.11)	44.92 (10.95)	46.23 (8.90)	49.67 (12.12)	45.24 (11.70)	47.31 (9.14)
RRS brooding	11.14 (3.18)	11.12 (3.14)	12.15 (3.54)	10.95 (3.57)	10.04 (2.85)	11.08 (3.62)	10.95 (3.57)	9.84 (2.87)	11.38 (2.93)
RRS reflection	10.86 (3.93)	10.40 (3.84)	10.58 (3.79)	10.52 (4.54)	9.88 (3.91)	10.77 (4.25)	10.67 (4.81)	10.32 (3.74)	10.77 (4.06)

#### Running Span Task

A 2×3 mixed anova was performed with R-Span Task score as dependent variable, time (pretraining and posttraining R-Span Task score) as within-subjects factor and training condition (dual n-back, single 1-back, or no training) as between-subjects factor. As expected there was a main effect of time, *F*(1, 69) = 6.80, *p* = .011, η^2^ = .090, indicating a better performance at posttraining (*M* = 21.83, *SD* = 5.46) compared to pretraining (*M* = 20.42, *SD* = 5.59). No main effect of training group was found, *F*(2, 69) = 1.68, *p* = .194, η^2^ = .046 and we failed to find the expected two-way interaction between time and training condition, *F*(2, 69) = 0.66, *p* = .522, η^2^ = .019. Controlling for baseline BDI-II and RRS scores did not alter the results.

#### Internal shift task

One participant did not complete the IST at pretraining, and was excluded for the IST analyses. A 2×3 mixed anova was performed with *emotional shift cost* as dependent variable, time (pretraining and posttraining) as within-subjects factor and training condition (dual n-back, single 1-back, or no training) as between-subjects factor. Unexpectedly, neither a main effect of time, *F*(1, 68) = 2.31, *p* = .13, η^2^ = .033, group, *F*(2, 68) = 1.19, *p* = .31, η^2^ = .020, nor a two way interaction between time and condition, *F*(2, 68) = 0.68, *p* = .51, η^2^ = .020 were found. The same analysis was performed with *non-emotional shift cost* as dependent variable. A main effect of time was found, *F*(1, 68) = 7.64, *p* = .007, η^2^ = .101, indicating a lower non-emotional shift cost posttraining (*M* = 356 ms, *SD* = 206 ms) compared to pretraining (*M* = 413 ms, *SD* = 208 ms). No main effect of group was found, *F*(2, 68) = 1.26, *p* = .29, η^2^ = .036 and also the two way interaction between time and condition was non-significant, *F*(2, 68) = 0.34, *p* = .71, η^2^ = .010. Controlling for baseline BDI-II and RRS scores did not alter the results.

#### Ruminative Response Scale

A 3×3 mixed anova was performed with time (pretraining, posttraining and follow-up RRS total score) as within-subjects factor and training condition (dual n-back, single 1-back, or no training) as between-subjects factor. Because sphericity could not be assumed, the *F* ratio was obtained applying the Huynh-Feldt correction [50]. No main time effect, *F*(1.79, 123.80) = 1.75, *p* = .18, η^2^ = .025, group effect, *F*(2, 69) = 0.73, *p* = .49, η^2^ = .021, or interaction effect, *F*(3.59, 123.80) = 0.29, *p* = .87, η^2^ = .008 were found.

The analysis was repeated with the brooding factor as dependent variable. A significant main effect of time was found, *F*(2, 138) = 6.28, *p* = .002, η^2^ = .089 (pretraining *M* = 11.50, *SD* = 3.29; posttraining *M* = 10.68, *SD* = 3.34; follow-up *M* = 10.72, *SD* = 3.14) but there was no significant group effect over time, *F*(2, 69) = 1.01, *p* = .37, η^2^ = .028 or time by condition interaction, *F*(4, 138) = 1.15, *p* = .34, η^2^ = .032.

When using the reflection factor as dependent variable, no significant main time effect, *F*(2, 138) = 0.51, *p* = .60, η^2^ = .007, group effect, *F*(2, 69) = 0.13, *p* = .88, η^2^ = .004, or time by condition interaction was found, *F*(4, 138) = 0.47, *p* = .76, η^2^ = .013. Controlling for baseline BDI-II score did not alter the results.

#### Beck Depression Inventory II

Because BDI-II scores were not normally distributed, a logarithmic transformation was performed, after increasing the scores with one point to avoid zero values. A 3×3 mixed anova was performed with BDI-II total score as dependent variable, time (pretraining, posttraining and follow-up BDI-II score) as within-subjects factor and training condition (dual n-back, single 1-back, or no training) as between-subjects factor. Because sphericity could not be assumed, the *F* ratio was obtained applying the Huynh-Feldt correction [50]. Neither a main time effect, *F*(1.67, 113.34) = 1.51, *p* = .23, η^2^ = .022, main effect of group, *F*(3, 68) = 0.21, *p* = .89, η^2^ = .009, nor interaction between time and condition, *F*(5, 113.34) = 1.39, *p* = .23, η^2^ = .058 were found.

## Experiment 1: Discussion

In experiment 1 we investigated whether working memory training had beneficial effects in individuals with high levels of rumination. For this purpose we compared individuals allocated to a working memory training with individuals who performed a less demanding one-back task or had a no task control condition. In contrast to the hypotheses, no differences between the experimental dual n-back training group and the control groups were found for the working memory tasks or the questionnaires. The training task performance of the dual n-back group improved over time, but we were not able to find measurable transfer effects of working memory training to other variables.

A second experiment was conducted to examine whether transfer effects of the same training paradigm could be observed on another set of basic and emotional working memory tasks. For this purpose we used the Operation Span Task [51] and the Emotional 2-back [20] task instead of the Internal Shift Task.

Another purpose of experiment 2 was to explore the potential moderating role of metacognitive beliefs on the effect of working memory training on rumination. It has been found that a subset of individuals with high levels of rumination hold positive or negative beliefs about rumination [52]. In our context, it is possible that individuals who hold negative beliefs about rumination will be the ones that benefit most from WM training because increased flexibility might help them to interrupt unwanted ruminative thinking. In contrast, individuals that hold positive beliefs about rumination could use the improved working memory capacity to ruminate more. Therefore, the role of metacognitions about rumination were also considered in experiment 2.

## Experiment 2: Methods

### Design overview

**Figure 3:**
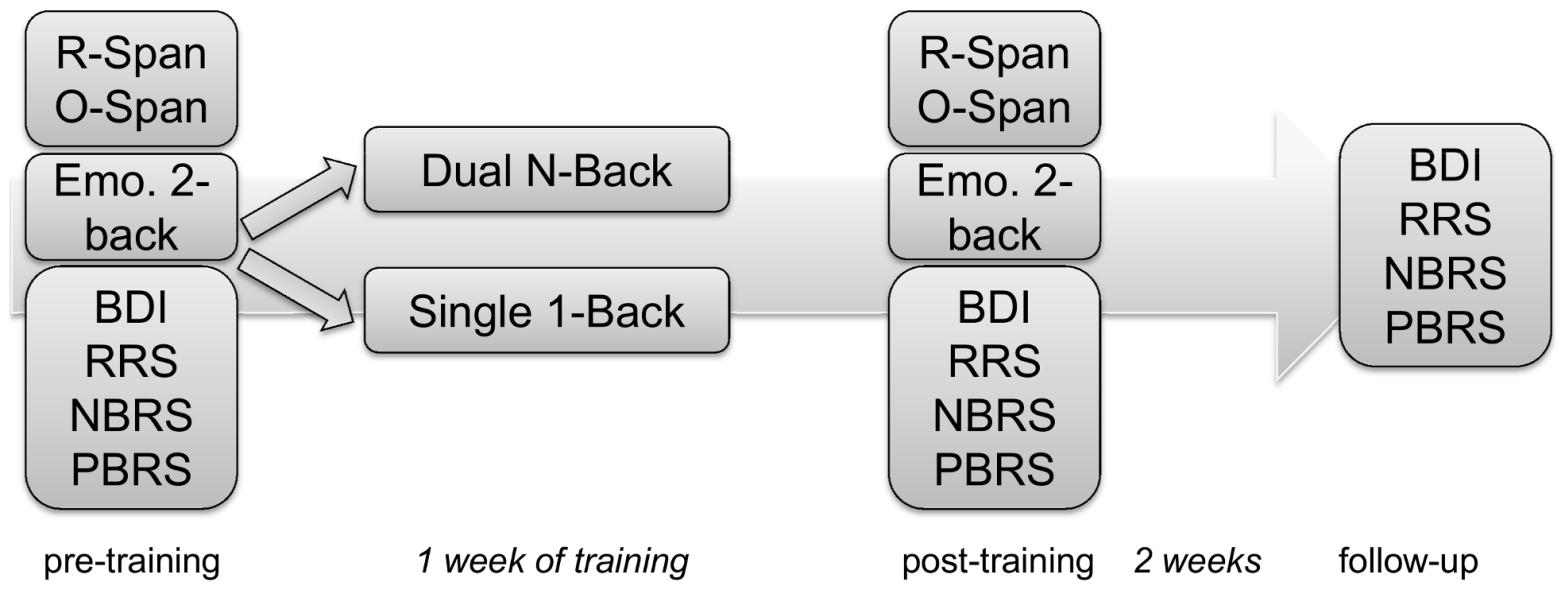
Design experiment 2.

An overview of the experiment design is presented in figure 3. At pre-training three working memory tasks, the Running Span Task, Operation Span Task and an emotional 2 back task were administered, followed by questionnaires. Subsequently, participants were randomly assigned to one of two homework training conditions. One week later the same computer tasks and questionnaires were administered at post-training. Another two weeks later, questionnaires were administered a third time during a follow-up.

### Participants

In this experiment, 53 undergraduates of Ghent University participated in return for a financial compensation (€ 30). The experiment was advertised through an online experiment managing system. Using the RRS as screening questionnaire, we tried to oversample participants scoring high on rumination.

Two participants were excluded because they did not show up for the posttest. One participant was excluded because she did not perform sufficient training sessions between both test moments. Another three participants were excluded because they did not complete the questionnaires. Based on outlier analysis, two more participants were excluded because of a high BDI-II score. This resulted in a final sample of 45 participants. Participants' characteristics are presented in table 4. Four participants did not complete the follow-up questionnaires. They were only excluded for the questionnaire analyses.

**Table 4:** Group characteristics.

	Dual n-back	Single 1-back
*N*	21	24
Age (*M* (*SD*))	20.76 (2.51)	21.54 (4.55)
Gender (M/F)	16/5	10/2

### Materials

#### Running memory Span Task

The R-Span Task used in this experiment was identical to the task described in the method section of experiment 1.

#### Operation Span Task

The Operation Span Task (O-span task) [53] was programmed by in line with Unsworth et al. [51] and ran using the Inquisit software package [42] on a Pentium 4 computer with a 60 Hz 17 inch CRT monitor. In the O-Span Task, participants were presented with mathematical problems they needed to solve, alternated with letters they had to remember. The actual task was preceded by three practice phases. In the first phase, three series of letters (a combination of F, H, J, K, L, N, P, Q, R, S, T, or Y) appeared on the screen at a rate of 800 ms per letter. Participants were instructed to recall the letters in the correct order. At the end of each series, participants could respond by clicking the recalled letters on a 4 ×3 matrix containing all possible letters. After the response, feedback was provided. In the second practice phase, 15 math problems (e.g. (8/2) + 9 = ?) were presented sequentially. Participants were instructed to solve the problems as quickly as possible, and to click the left mouse button as soon as they knew the answer. After clicking, a number was presented on the screen and participants had to judge whether this was the correct solution by clicking *true* or *false*. After each math problem, accuracy feedback was provided. In the third practice phase solving math problems and recalling letters was combined. First, participants saw a math problem. Next, after clicking to indicate they had solved the problem and subsequently judging whether the presented number was correct, a letter was presented for 800 ms, after which a new math problem and letter combination appeared. During the second practice phase, the participant's reaction time to indicate the solution had been found, was recorded. The mean reaction time plus 2.5 standard deviations was used in the third practice phase as a time limit for the mathematical problem, to prevent participants from rehearsing the letters while solving the mathematical problem. If reaction time exceeded this time limit, the letter was shown immediately and the response to the mathematical problem was coded as a speed error. At the end of each series, participants had to report the recalled letters on a 4×3 matrix. During the third practice phase, three series of two mathematical problem and letter combinations were presented.

The instructions for the actual task were identical to the third practice phase. The length of each series was ranging from three to seven, and during the O-Span Task each length was presented three times, in a random order. So 15 series were presented, comprising 75 mathematical problems and 75 letters. Participants were encouraged to keep the overall mathematical problem accuracy above 85%, in order to avoid participants focusing only on remembering the letters. After each series, the overall accuracy percentage was presented during feedback. The main outcome variable used in the analyses was the *O-Span Task score*, being the sum of all letters in perfectly recalled series.

#### Emotional 2 back task

The emotional 2-back task was adapted from Levens and Gotlib [20] and programmed using the E-prime software package [43]. It ran on the same computer as the R-Span Task and O-Span Task. In the emotional 2-back task, words were presented sequentially and participants had to judge whether the current word was identical (match trial; e.g. … flower - closet - water - closet) or different (mismatch trial; e.g. … flower - death - water - closet) to the word presented two steps before, by pressing “G” or “H” respectively. Each word was presented for 1000 ms in the center of the screen with an interstimulus interval of 1000 ms. The task consisted of six blocks, each containing 47 words. The order of the blocks was randomized, but within the blocks there was a fixed random, to ensure sufficient observations of the word combinations of interest. The blocks were preceded by a training block of 15 words that was excluded in further analyses. All words were taken from the Dutch affective word norms database [54] of which 214 were neutral (mean valence rating), 33 positive (> 3 SD above mean valence rating) and 35 negative (> 3 SD below mean valence rating). All words scored average on arousal and power ratings and had one or two syllables.

Because the original task by Levens and Gotlib [20] was too time-consuming to include in the study design, only the most relevant trial types were retained to reduce the number of trials. More specifically this experiment only examined lure trials [55], [56]. This is a mismatch trial in which not the relevant word (two positions before) but the word one (n-1 lure trial; e.g. … flower - water - *death* - death), three (n+1 lure trial; e.g. … *death* - water - flower - death) or four (n+2 lure trial; e.g. … *death* - grass - water - flower - death) positions before is identical to the current word. These lure trials are a measure of interference of positive versus negative words in the continuous updating process. In other words, n+1 and n+2 lure trials measure the interference of no longer relevant information, while the n-1 lure trial measures the interference of not yet relevant information.

In sum, 89 match and 181 mismatch trials were presented. The first two trials of each block did not require a response. Both reaction time and accuracy of each response were recorded. For every lure trial type, a difference in mean reaction time between negative and positive lure trials could be calculated, indicating the difference between interference of positive and negative information.

#### Dual n-back, sound 1-back and position 1-back

All tasks used during the one week training period were identical to the tasks described in the method section of experiment 1.

#### Questionnaires

Next to the BDI-II and RRS, that were identical to the versions described in experiment 1, the Dutch translations of the Positive Beliefs about Rumination Scale (PBRS) [57], [58] and the Negative Beliefs about Rumination Scale (NBRS) [52], [58] were administered. The PBRS is a nine item questionnaire assessing positive metacognitive beliefs about the possible benefits of rumination, while the NBRS is a 13 item questionnaire assessing negative metacognitive beliefs about rumination. The NBRS consist out of two subscales, an eight item subscale that assesses metacognitive beliefs about uncontrollability and harm associated with rumination and a five item subscale that assesses metacognitive beliefs about interpersonal and social consequences of rumination [59]. Both questionnaires are scored on a four-point Likert scale ranging from 1 (do not agree) to 4 (agree very much).

### Procedure

Most procedural elements were kept similar to Experiment 1 to allow comparison. Participants were given limited instructions about the purpose of the study. They were told their participation consisted out of a six day homework computer task, preceded and followed by three computer tasks in the lab and some questionnaires. All participants provided their written informed consent to participate in this study. At pretest, the R-Span Task, O-Span Task and emotional 2 back task were administered after completing the informed consent form. The BDI-II and RRS, NBRS and PBRS had to be completed the same day. Participants were randomly allocated to the dual n-back training condition or single 1-back control condition. Within the control-training condition, participants were randomly allocated to either the position or the sound 1-back control training. Participants in the position and sound 1-back condition were combined to one single 1-back control condition. After exactly one week of daily training, both computer tasks and questionnaires were administered at posttest. Finally, two weeks after posttest, the questionnaires were completed as follow-up. At the end of this follow-up, participants were debriefed about the purposes of this experiment. This experiment was approved by the ethics committee of the Faculty of Psychology and Educational Sciences, Ghent University.

### Statistical analyses

Associations between computer task scores and questionnaires were inspected using Pearson correlations. To test for baseline differences between groups, a set of one way anovas was executed with group as independent variable. The effect of training was tested using a mixed anova design for each outcome variable, with time as within subject factor and group as between subject factor. To test for the effect of beliefs about rumination, both positive and negative beliefs about rumination were added as covariates to the mixed anovas with rumination scores as outcome. Next the mixed anovas were performed in a subgroup with relatively high negative beliefs about rumination.

## Experiment 2: Results

### Baseline correlations

Correlations between the baseline computer tasks and questionnaires are presented in table 5. Running and operation span scores were significantly correlated. However, only operation span score was correlated with ruminative brooding. The emotional 2-back variables were not significantly correlated with the other computer tasks or questionnaires, except for the n+2 lure difference score that was negatively correlated with BDI-II score. BDI-II score was correlated with RRS brooding score and positive beliefs about rumination. RRS total score was correlated with both RRS factors and both positive and negative beliefs about rumination. Brooding score was correlated with negative beliefs about rumination and reflection score was correlated with both positive and negative beliefs about rumination. Finally, positive and negative beliefs about rumination were correlated.

**Table 5:** Pearson correlations between baseline variables.

	(1)	(2)	(3)	(4)	(5)	(6)	(7)	(8)	(9)	(10)	(11)
(1) R-Span Task score	-	.433^**^	.196	.016	.006	−.178	.273	.298	.084	−.037	−.068
(2) O-Span Task score		-	−.075	−.017	−.025	−.102	.281	.343*	.214	.001	−.151
(3) Lure n-1 diff.			-	.259	−.235	.241	−.014	.003	.069	−.017	.055
(4) Lure n+1 diff.				-	−.023	−.016	−.177	−.220	.095	−.183	.093
(5) Lure n+2 diff.					-	−.298*	−.026	−.033	−.080	−.133	−.108
(6) BDI-II total						-	.188	.298*	.092	.273	.376*
(7) RRS total							-	.826^**^	.551^**^	.532^**^	.460^**^
(8) RRS brooding								-	.230	.379*	.209
(9) RRS reflection									-	.363*	.382^**^
(10) NBRS										-	.406^**^
(11) PBRS											-

^*^ *p*<.05. ^**^ *p*<.01

### Baseline group differences

Independent sample *t* tests indicated that mean age, R-Span Task, emotional 2 back, BDI-II, RRS, NBRS and PBRS scores were not significantly different between the dual n-back and single 1-back control condition (all *t*s <1.9) at pretest. Also gender ratio did not differ between conditions, χ^2^(1, *N* = 45) = 0.009, *p* = .926 (see table 2). However, the dual n-back group had a significantly higher pretest O-Span Task score (*M* = 47.33, *SD* = 16.48) than the single 1-back control group (*M* = 35.88, *SD* = 18.41), *t*(43) = 2.187, *p* = .034, Cohen's *d* = 0.68.

### Dual n-back training performance

**Figure 4:**
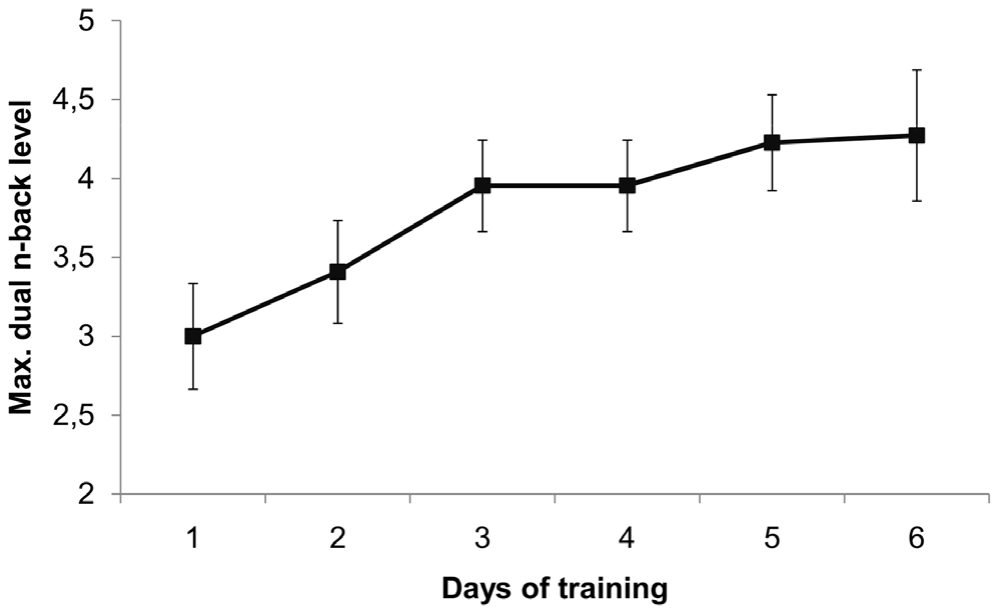
Dual n-back progress with 95% confidence intervals for experiment 2.

During the six day training, the performance of the adaptive dual n-back training group improved significantly. The average daily maximum *n*-level increased gradually from 3.00 (*SD* = 0.71) at day one to 4.38 (*SD* = 0.97) at day six, *t*(20) = 5.66, *p*<.001, Cohen's *d* = 1.62 (see figure 4). The increase in maximum n-level was not significantly correlated with the difference scores (post- minus pre-training score) for the R-Span, emotional 2-back, RRS and BDI-II, all *p*'s >.17. There was however a marginally significant correlation between dual n-back improvement and O-span difference score, *r* = .415, *p* = .061, indicating larger training gains are marginally associated with a larger increase in O-span performance.

### Analyses of transfer effects

All task and questionnaires score are presented in table 6. Sound 1-back and position 1-back participants were collapsed after data exploration indicated no differences for all analyses.

**Table 6:** Computer task and questionnaire scores for T1, T2 and T3.

	Pre-training (T1)		Post-training (T2)		Follow-up (T3)	
	Dual n-back	Single 1-back	Dual n-back	Single 1-back	Dual n-back	Single 1-back
R-span	19.76 (5.94)	18.54 (5.63)	22.14 (5.93)	21.29 (7.27)	-	-
O-span	47.33 (16.48)	35.88 (18.41)	54.95 (12.77)	44.71 (15.71)	-	-
Emotional 2-back						
Lure n-1 diff. (ms)	0 (211)	38 (224)	1 (187)	-17 (88)	-	-
Lure n+1 diff. (ms)	46 (187)	130 (181)	26 (159)	35 (109)	-	-
Lure n+2 diff. (ms)	2 (176)	14 (208)	28 (118)	9 (152)	-	-
BDI-II	5.58 (4.65)	4.82 (4.62)	6.21 (4.42)	4.32 (4.11)	6.74 (6.58)	3.95 (4.49)
RRS total	43.95 (11.64)	37.45 (10.31)	44.37 (11.09)	36.73 (9.37)	45.37 (13.78)	36.09 (9.99)
RRS brooding	10.95 (3.69)	8.64 (2.82)	10.68 (3.54)	8.41 (2.38)	11.32 (4.07)	8.59 (3.05)
RRS reflection	9.00 (3.58)	7.55 (2.26)	8.68 (3.47)	7.77 (2.56)	8.84 (3.44)	7.45 (2.24)

#### Running Span Task

A 2×2 mixed anova was performed with R-Span Task score as dependent variable, time (pretraining and posttraining R-Span Task score) as within-subjects factor and training condition (dual n-back or single 1-back) as between-subjects factor. As expected there was a main effect of time, *F*(1, 43) = 8.42, *p* = .006, η^2^ = .16, indicating a better performance at posttraining (*M* = 21.71, *SD* = 0.99) compared to pretraining (*M* = 19.15, *SD* = 0.86). The difference between both training groups over time was non significant, *F*(1, 43) = 0.40, *p* = .532, η^2^ = .009 and we failed to find the hypothesized two-way interaction between time and training condition, *F*(1, 43) = 0.044, *p* = .836, η^2^ = .001. In sum, the increase in R-Span Task performance did not differ significantly between groups. Controlling for baseline BDI-II and RRS scores did not alter the results.

#### Operation Span Task

A 2×2 mixed anova was performed with O-Span Task score as dependent variable, time (pretraining and posttraining O-Span Task score) as within-subjects factor and training condition (dual n-back or single 1-back) as between-subjects factor. As expected there was a main effect of time, *F*(1, 43) = 13.57, *p* = .001, η^2^ = .24, indicating a better performance at posttraining (*M* = 49.83, *SD* = 2.15) compared to pretraining (*M* = 41.60, *SD* = 2.15). Regardless of time, the training group (*M* = 51.14, *SD* = 3.10) had a significantly higher O-Span Task score than the control group (*M* = 40.29, *SD* = 2.90), *F*(1, 43) = 6.54, *p* = .014, η^2^ = .132. Finally, we failed to find the expected two-way interaction between time and training condition, *F*(1, 43) = 0.074, *p* = .787, η^2^ = .002. So the increase in O-Span Task performance did not differ significantly between groups. Controlling for baseline BDI-II and RRS scores did not alter the results.

#### Emotional 2 back

A 2×2 mixed anova was performed with the difference between mean reaction time for negative and positive n-1 lure trials as dependent variable, time (pretraining and posttraining) as within-subjects factor and training condition (dual n-back or single 1-back) as between-subjects factor. There was no significant effect of time, *F*(1, 41) = 0.41, *p* = .526, η^2^ = .010, or training group, *F*(1, 41) = 0.070, *p* = .793, η^2^ = .002. Also the expected two way interaction between time and group was not significant, *F*(1, 41) = 0.42, *p* = .523, η^2^ = .010.

The same analysis was repeated with the difference between mean reaction time for negative and positive n+1 lure trials as dependent variable. Similar to the previous analysis, no significant main effect of time, *F*(1, 41) = 2.74, *p* = .106, η^2^ = .063, training group, *F*(1, 41) = 1.78, *p* = .190, η^2^ = .042, or time by group interaction effect, *F*(1, 41) = 1.17, *p* = .285, η^2^ = .028, was found.

Comparable results were found when using the difference between mean reaction time for negative and positive n+2 lure trials as dependent variable. No significant main effect of time, *F*(1, 42) = 0.86, *p* = .771, η^2^ = .002, training group, *F*(1, 42) = 0.011, *p* = .918, η^2^ = .000, or time by group interaction effect, *F*(1, 42) = 0.164, *p* = .688, η^2^ = .004, was found. Controlling for baseline BDI-II and RRS scores did not alter the results.

#### Ruminative Response Scale

A 3×2 mixed anova was performed with RRS total score as dependent variable, time (pretraining, posttraining and follow-up) as within-subjects factor and training condition (dual n-back or single 1-back) as between-subjects factor. Because sphericity could not be assumed, the *F* ratio was obtained applying the Huynh-Feldt correction [50]. No main time effect, *F*(1.87, 72.84) = 0.029, *p* = .972, η^2^ = .001, was found. However, the dual n-back group had a significantly higher score (*M* = 44.56, *SD* = 2.44) than the single 1-back control group (*M* = 36.758, *SD* = 2.26), *F*(1, 39) = 5.508, *p* = .024, η^2^ = .124, no group by time interaction, *F*(1.87, 70.68) = 1.475, *p* = .236, η^2^ = .036 were found.

The same analysis was performed with the brooding factor as dependent variable. A significant main effect of group was found, *F*(1, 39) = 6.39, *p* = .016, η^2^ = .141 (dual n-back *M* = 10.98, *SD* = 0.706; single 1-back *M* = 8.55, *SD* = 0.656) but there was no significant time, *F*(2, 78) = 0.938, *p* = .396, η^2^ = .023, or time by condition interaction effect, *F*(2, 78) = 0.349, *p* = .706, η^2^ = .009.

Using the reflection factor as dependent variable, no time, *F*(2, 78) = 0.198, *p* = .821, η^2^ = .005, group, *F*(1, 39) = 1.975, *p* = .168, η^2^ = .048, or time by group interaction effect, *F*(2, 78) = 1.092, *p* = .341, η^2^ = .027, was found. Controlling for baseline BDI-II score did not alter the results of all rumination analyses.

#### Effects of negative and positive beliefs about rumination

Adding negative and positive beliefs about rumination as covariates did not alter the crucial interactions in the mixed anovas with rumination scores as outcome.

In a next step, median split was used to select participants with a relatively high pre training total NBRS score. In this subgroup, 13 participants received the dual n-back training, and 10 participants received a single 1-back control training.

A 3×2 mixed anova was performed with RRS total score as dependent variable, time (pretraining, posttraining and follow-up) as within-subjects factor and training condition (dual n-back or single 1-back) as between-subjects factor. No main time effect, *F*(2, 38) = 0.310, *p* = .736, η^2^ = .016 was found. The dual n-back group had a significantly higher score (*M* = 50.75, *SD* = 2.48) than the single 1-back control score (*M* = 41.33, *SD* = 2.86), *F*(1, 19) = 6.196, *p* = .022, η^2^ = .246 but no group by time interaction, *F*(2, 38) = 1.824, *p* = .175, η^2^ = .088 was found.

The same analysis was performed with the brooding factor as dependent variable. A significant main effect of group was found, *F*(1, 19) = 7.11, *p* = .015, η^2^ = .272 (dual n-back *M* = 12.67, *SD* = 0.87; single 1-back *M* = 9.11, *SD* = 1.01) but there was no significant time, *F*(2, 38) = 0.809, *p* = .453, η^2^ = .041, or time by condition interaction effect, *F*(2, 38) = 0.582, *p* = .563, η^2^ = .030.

Using the reflection factor as dependent variable, no time, *F*(2, 38) = 0.633, *p* = .537, η^2^ = .032, group, *F*(1, 19) = 0.193, *p* = .665, η^2^ = .010, or time by group interaction effect, *F*(2, 38) = 1.039, *p* = .363, η^2^ = .052, was found. Controlling for baseline BDI-II score did not alter the results.

#### Beck Depression Inventory II

Because BDI-II scores were not normally distributed, a logarithmic transformation was performed, after increasing the scores with one point to avoid zero values. A 3×2 mixed anova was performed with BDI-II total score as dependent variable, time (pretraining, posttraining and follow-up BDI-II score) as within-subjects factor and training condition (dual n-back or single 1-back) as between-subjects factor. Neither a main time effect, *F*(2, 78) = 0.17, *p* = .844, η^2^ = .004, main effect of group, *F*(1, 39) = 2.199, *p* = .146, η^2^ = .053, nor interaction between time and condition, *F*(5, 113.34) = 1.565, *p* = .216, η^2^ = .039 were found.

## Experiment 2: Discussion

Comparable to experiment 1, no differences between the dual n-back training group and the control group were found. Adapting the set of working memory tasks did not change the outcome of the experiment. Furthermore, no differential training effects on rumination scores were found for the subgroup scoring high on negative beliefs about rumination. Possible explanations for the failure to find training effects are discussed below.

## General Discussion

In the present study, we examined the effects of a working memory training on working memory functioning and depressive symptoms, in particular rumination. The motivation for this study was twofold. First, using a cognitive bias modification approach, the causality of the association between working memory, rumination and subclinical depressive symptomswas examined. To expand our knowledge about factors that causally contribute to the onset or maintenance of depression, this kind of experimental research is required. Second, this study provides further insights in the clinical utility of online working memory training in reducing levels of rumination and depressive symptoms.

The results of both experiments did not confirm our three main hypotheses. There was no difference in the evolution of (1) non-emotional and (2) emotional working memory performance and (3) rumination and depression scores, regardless of metacognitive beliefs about rumination between the experimental dual n-back group and the control groups after one week of training. In other words, the gain in dual n-back performance did not transfer to our outcomes. In terms of the cognitive bias modification approach, no evidence was found for a clear causal role of working memory. These results also show no evidence to use one week of dual n-back training as a real life intervention.

Before drawing further conclusions on these results, it is important to start considering the validity and reliability of these null-findings. There are several features of the present study that attest to its validity. First, the present study used an experimental design in which all participants were randomly assigned to either the experimental, active control or passive control condition. Second we used a training task that has proven to be effective [32] and only included participants who performed this training the required number of times. Third, a set of validated working memory tasks, each tapping another aspect of working memory and questionnaires were used as outcome, which has been considered as one of the crucial methodological desiderata in this type of research [31]. Furthermore, reliability is demonstrated by obtaining the same findings in experiment 1 and 2.

Several explanations could account for the absence of effects of working memory training. As already mentioned, finding meaningful transfer effects is one of the key challenges of working memory training research [28]–[31]. Moreover in this study the number of training sessions was confined to 6 days of twenty minutes working memory training. This might be too limited to find measurable transfer effects. The number of training days in the study of Jaeggi et al. [32] ranged from 8 to 19, and they only found transfer effects to fluid intelligence measures after 17 days of training. This indicates that transfer effects depend on the dosage of the training. However, the number of days required to obtain transfer to fluid intelligence cannot be generalized to the number of days required to find transfer to working memory functioning with non-emotional and emotional information and depression and rumination measures. For instance, in the study by Owens et al. [37] transfer on a cognitive task (tapping visual working memory) was observed after eight training sessions.

A major methodological issue in training studies is the use of an adequate control group [30], [31]. Especially there has been criticism on previous studies where an active control group was lacking. Therefore, we opted to include in each experiment an active control task that only differed in the load imposed on working memory. One can argue that possible training effects were masked because this active control training also improved working memory processes. Therefore the analyses in Experiment 1 were repeated comparing the dual n-back training with each control group separately, which did not change the results. Moreover, previous work also indicated that differences can be observed between the dual n-back training and the 1-back control task that was used [37].

Another important issue to consider is statistical power. Based on the large effect size (Cohens' *d* = 0.9) found in the study of Owens et al. [37] using the same training task, we calculated the sample size needed to demonstrate an effect with a similar effect size using G*Power [60]. This analysis indicated that a total sample size of 21 for every condition is required with α = .95 and β = .80. In both studies, this minimum is obtained, so the current null-findings cannot be attributed to a lack of power.

What to make of the current null-findings? It is noteworthy to mention that there is quite an extensive number of studies that have failed to observe any benefits from working memory training in healthy, non-clinical samples [31]. This contrasts with other studies where working memory training has been successful in individuals with higher levels of psychopathology. For instance, in the study by Owens et al. [37] participants were selected on high levels of depressive symptoms. Another study found that working memory training has beneficial effects in alcoholism [36]. In this study, participants were selected on heightened rumination scores. However, the samples still consisted out of high-level university students. In this specific sample, there was a lack of correlations between working memory task performance and rumination scores at baseline. Hence, it may be that working memory training is only capable of improving cognitive functioning and psychopathology if there are severe cognitive impairments at the onset. Therefore, the current null findings are crucial in determining the conditions when training is and is not effective. Therefore, training studies using clinical samples with a clear presence of cognitive impairments are needed.

In relation to rumination, we need to be cautious in concluding that working memory processes play no causal role in rumination and depressive symptoms. Provided that no transfer of training was found to the cognitive processes underlying rumination, we were unable to investigate the effect of improved cognitive functioning on the tendency to ruminate. Lack of transfer could be due to the training having no substantial effect in a non-clinical group with only mild cognitive symptoms of depression.

One interesting direction for future research would be to examine whether emotional variants of n-back training have more effect on changing affective working memory processes and rumination. In this domain, the work of Schweizer et al. [61] is interesting where they showed that only an emotional variant and not the standard version of the dual n-back training led to transfer effects on affective emotional processing. In a recent study Schweizer et al. [62] demonstrated that compared to a placebo control training, 20 days of emotional dual n-back training resulted in improved emotion regulation. Furthermore, fMRI measures indicated that this improvement was associated with increased activation of brain regions involved with mood regulation.

There are some limitations to the present study. In both experiments we used an undergraduate sample with heightened rumination score and a majority of female participants. Therefore results might not be generalizable to a community or clinical sample. University students may be characterized by relatively good cognitive functioning, which might make it more difficult to obtain a substantial gain in cognitive performance due to ceiling effects. Second, the number of training days was limited to six. Increasing the duration and/or intensity of the training might lead to measurable transfer effects. Third, there were no pre- and post training measures of dual n-back performance. Therefore we could not monitor if the low intensity training also led to improvements. However, we specifically decided not to include pre- and post-measures of dual n-back performance, because we rather wanted to examine transfer effects and we did not want to expose the control group to multiple dual n-back task administrations (that could potentially also improve their working memory performance). Finally, even though we tried to use valid paradigms, it is possible that the current set of working memory tasks does not capture the affected working memory processes in high ruminators sufficiently. Alternative tasks, measuring different aspects of working memory functioning might lead to other results.

In sum, the current study failed to find evidence for transfer effects of a six day non-emotional dual n-back training on emotional and non-emotional working memory performance, and measures of rumination and depressive symptoms. Recent studies express the need for methodologically sound research to demonstrate transfer of training effects and the mechanisms underlying this transfer. Moreover, interesting results have been found using an emotional variant of working memory training. Therefore we hope that this study invites to further investigate the possibility of a working memory training paradigm that directly targets the processing of emotional information in a population with a cognitive vulnerability for depression.
